# Assessment on Potential Suitable Habitats of the Grasshopper *Oedaleus decorus asiaticus* in North China based on MaxEnt Modeling and Remote Sensing Data

**DOI:** 10.3390/insects14020138

**Published:** 2023-01-29

**Authors:** Zhongxiang Sun, Huichun Ye, Wenjiang Huang, Erden Qimuge, Huiqing Bai, Chaojia Nie, Longhui Lu, Binxiang Qian, Bo Wu

**Affiliations:** 1 China Agricultural Museum, Beijing 100125, China; 2 State Key Laboratory of Remote Sensing Science, Aerospace Information Research Institute, Chinese Academy of Sciences, Beijing 100094, China; 3 Key Laboratory of Earth Observation of Hainan Province, Sanya 572029, China; 4 University of Chinese Academy of Sciences, Beijing 100049, China; 5 Grassland Workstation of Xilinguole League, Inner Mongolia Autonomous Region, Xilinguole League 026000, China; 6 Institute of Environment and Sustainable Development in Agriculture, Chinese Academy of Agricultural Sciences, Beijing 100081, China

**Keywords:** grasshopper, habitat factor, suitable area, remote sensing, MaxEnt modeling, agricultural heritage systems

## Abstract

**Simple Summary:**

*Oedaleus decorus asiaticus* (Bey-Bienko) populations are highly concentrated in Xilingol League of the Inner Mongolia Autonomous Region where they regularly harm the ecosystems protecting northern China. In order to prevent the occurrence of *O. d. asiaticus* in the Xilingole grassland, the establishment of a high-precision method and extracting the area of occurrence for *O. d. asiaticus* is required. The primary objectives of this study were: (1) to project the potential distribution of *O. d. asiaticus* by MaxEnt model and remote sensing data, and (2) to explore the responses and relative contributions of habitat factors to the distribution of *O. d. asiaticus*. The study will help guide managers and decision-makers to prevent and control the occurrence of *O. d. asiaticus* early on and this work may facilitate meaningful reductions in pesticide application.

**Abstract:**

Grasshopper populations can quickly grow to catastrophic levels, causing a huge amount of damage in a short time. *Oedaleus decorus asiaticus* (Bey-Bienko) (*O. d. asiaticus*) is the most serious species in Xilingol League of the Inner Mongolia Autonomous Region. The region is not only an important grassland but also a site of agricultural heritage systems in China. Therefore, projecting the potential geographic distribution of *O. d. asiaticus* to provide an early warning is vital. Here, we combined temperature, precipitation, soil, vegetation, and topography with remote sensing data to screen the predictors that best characterize the current geographical distribution of *O. d. asiaticus*. A MaxEnt model approach was applied to project the potential suitable distribution of *O. d. asiaticus* in Xilingol League (the Inner Mongolia Autonomous Region of China) combined with a set of optimized parameters. The modeling results indicated that there were six main habitat factors that determined the suitable distribution of *O. d. asiaticus* such as the soil type (ST), grassland type (GT), elevation, precipitation during the growing period (GP), precipitation during the spawning period (SP), and normalized difference vegetation index during the overwintering period (ONDVI). The simulated result was good, with average AUC and TSS values of 0.875 and 0.812, respectively. The potential inhabitable areas of grasshoppers were 198,527 km^2^, distributed mainly in West Urumqi, Xilinhot City, East Urumqi, Abaga Banner, and Xianghuang Banner of Xilingol League. This study is valuable to guide managers and decision-makers to prevent and control the occurrence of *O. d. asiaticus* early on and this study may facilitate meaningful reductions in pesticide application.

## 1. Introduction

The *Oedaleus decorus asiaticus* (*O. d. asiaticus*) populations are highly concentrated in Xilingol League of the Inner Mongolia Autonomous Region where they regularly harm the ecosystems protecting northern China. Moreover, previous research has explored the relationships between *O. d. asiaticus* and climate and showed that the *O. d. asiaticus* would occur with a high density since the El Niño phenomenon happens [1,2]. Therefore, the control of *O. d. asiaticus* is not only valuable for the construction of ecological grasslands but it is also vital to maintaining healthy grass ecosystems and supporting the livestock depending on grass.

Disasters caused by locusts and grasshoppers have occurred in China over thousands of years. Analyzing the occurrence patterns of locusts and grasshoppers and their drivers is the basis of related prediction researches [3,4]. Previous studies have concluded that the relationships were close and complex between a locust occurrence and various ecological factors [5,6,7,8,9]. Habitat factors (e.g., meteorology, topography, physical and chemical properties of soil, ecogeographic features, etc.) influence the growth, development, and community structure of locusts and grasshoppers [10,11,12,13,14,15,16]. Among the meteorology factors, temperature and precipitation are two influence factors which affect the growth of locusts and grasshoppers directly and significantly [10,11,12,17]. The impact of precipitation on the density of locusts and grasshoppers varies among different regions [18,19]. Except the meteorological factors, locusts and grasshoppers are influenced by the changes in land use and the soil temperature [13,14]. The hatching rate of locusts and grasshoppers has been proven to raise with an increasing soil temperature [20]. Moreover, the physical and chemical properties of soil (soil texture, soil moisture, pH, salinity, etc.) also affect the growth and development of locusts and grasshoppers [19]. Topography, including landform type, elevation, slope, and orientation, indirectly affect the distribution and density of locusts and grasshoppers by redistributing the temperature, precipitation, and light [16]. The hatching rate of locusts and grasshoppers increased with an increasing soil temperature [20]. Furthermore, habitat factors indirectly affect the locust and grasshopper community and its emergence dynamics by influencing the growth and distribution of the plant community in their habitats [21]. Locusts and grasshoppers are suited to survive in environments with homogeneous vegetation and a variable climate. As a key ecological factor, vegetation not only provides food resources but also represents a suitable habitat for locusts and grasshoppers [22], significantly affecting the occurrence of locust and grasshopper disasters. In general, these habitat factors modify the plant community, influencing its growth and distribution, and therefore affect the emergence dynamics of locusts and grasshoppers.

The methods for extracting the habitat factors mainly consist of species distribution models and expert empirical scoring. However, the expert empirical method is inadequate in the quantification of indicators due to the subjective experiences of researchers [23]. The species distribution model is a mathematical method for the unbiased inference of unknown distributions based on limited information [24]. MaxEnt performs well in modeling species niches, even though there are limited occurrence data [25]. Moreover, the MaxEnt model can screen and quantify habitat factors well. Traditional methods of extracting locusts and grasshoppers’ habitat have focused on ground surveys and meteorological information in China; however, the representation of the data obtained is low due to sparse grassland areas and the limited number of monitoring points [26,27]. In recent years, remote sensing (RS) technology, as a new means of obtaining continuous, large-scale spatial and temporal information on the Earth’s surface, has been widely used for monitoring and assessing locust and grasshopper disasters. A large number of scholars have applied remote sensing technology to explore the remote inversion of grassland habitat factors, mechanisms of locusts and grasshoppers’ occurrence related to habitats, and the prediction of locusts and grasshoppers’ occurrence areas and levels [28,29,30,31]. There are multiple habitat factors that affected locusts’ growth and selecting suitable indicators for modeling is a vital step that directly determines the precision of predicting the occurrence of locusts and grasshoppers. However, previous studies are mainly based on a single meteorological or remote sensing factor and do not consider the different reproductive periods of locusts and grasshoppers [32].

**Figure 1:**
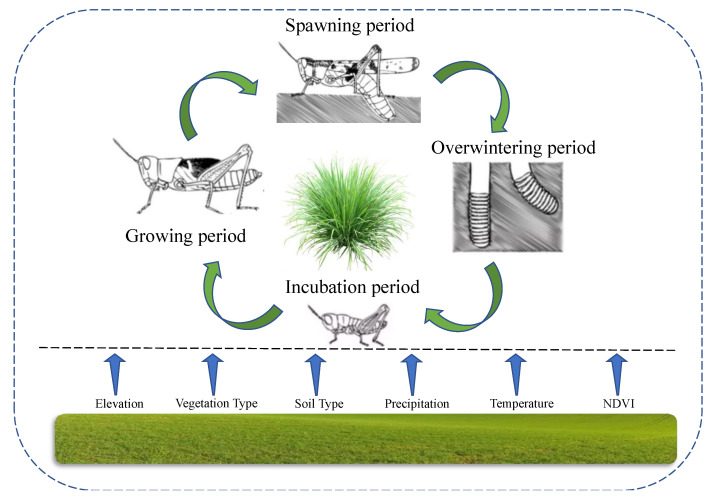
The environmental factors and reproductive periods of *O. d. asiaticus*.

The main species of grasshoppers in Xilingol League are *O. d. asiaticus*, *Dasyhippus barbipes* (Fischer—Waldheim), and *Bryodema luctuosum*. Here, the *O. d. asiaticus* was selected due to the most serious species of disaster. *O. d. asiaticus* eggs are laid from July to September, remain in the soil during the cold winter period (October–March), before hatching during the period of April–May in the following year, and finally grow to maturity in June. Therefore, we divided the reproductive periods into four stages, such as the spawning period (July–September 2019), overwintering period (January–February 2020), incubation period (April–May 2020), and growing period (June 2020). Note that these stages are correspond to the habits of *O. d. asiaticus* (Figure 1).

In summary, in order to prevent the occurrence of grasshoppers in the Xilingole grassland, a high-precision method needs to be established and a suitable area of the occurrence of *O. d. asiaticus* needs to be extracted. It is valuable for facilitating large-scale quantitative monitoring and developing efficient management strategies for *O. d. asiaticus*. The primary objectives of this study were: (1) to select a MaxEnt model with the optimal combination of parameters to best predict the potential distribution of *O. d. asiaticus*; (2) to explore the responses and relative contributions of habitat factors to the distribution of *O. d. asiaticus*; and (3) to characterize the areas in Xilingole, which are suitable for *O. d. asiaticus*. The study can guide managers and decision-makers in providing an early warning for the disaster occurrence of *O. d. asiaticus* and may further help the precise control by using pesticides and reducing the amount of pesticide application.

## 2. Materials and Methods

### 2.1. Study Area and Species

**Figure 2:**
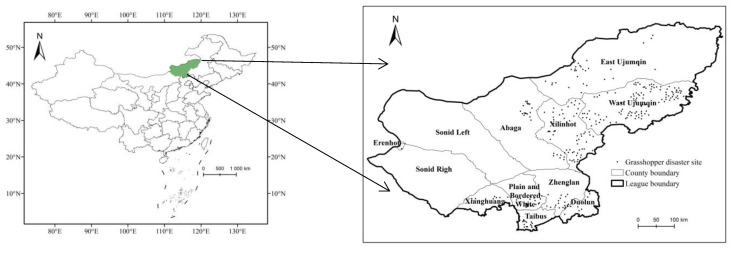
Experimental area location and field survey sampling positions of *O. d. asiaticus*.

The *O. d. asiaticus* populations are concentrated in Xilingol League of China, containing existing areas of grasshoppers’ occurrence of nearly 300 km^2^ across 13 banners (counties, cities, and districts). Hundreds of tons of chemical pesticides have been applied to prevent and control *O. d. asiaticus* disasters in this region [2]. In 2020, *O. d. asiaticus* damaged grasslands in an area of 235.10 km^2^ in the Xilingol League, mainly distributed in the West Ujimqin Banner, Xilinhot city, East Ujimqin Banner, and Abaga Banner (Figure 2).

The *O. d. asiaticus* occurrence data used in this study were provided by the grassland workstation of Xilingol League, Inner Mongolia Autonomous Region (http://www.forestry.gov.cn/, accessed on 30 August 2020). *O. d. asiaticus* surveys were conducted from June to August 2020. If the grasshopper densities were above 15 specimens per square meter in the grassland, the location was marked as a grasshopper distribution site, and a total of 310 occurrence points were collected (Figure 2).

### 2.2. Habitat Factors

Based on the biological characteristics of *O. d. asiaticus*, this study utilized four categories of environmental variables, including soil, vegetation, topography, and meteorology, to establish a model for assessing the potential geographic distribution of the *O. d. asiaticus* (Table 1).

The soil indicator (soil type data) was obtained from the 1:1 million China soil map (https://www.resdc.cn/, accessed on 12 April 2019). The topography indicator (elevation data) was derived from the ASTER GDEM V3 data, obtained from China’s Geospatial Data Cloud (http://www.gscloud.cn/, accessed on 5 April 2020).

The vegetation indicators, including the vegetation type and normalized difference vegetation index (NDVI), were obtained from China’s Resource and Environment Science and Data Center (https://www.resdc.cn/, accessed on 21 June 2019). To describe the vegetation condition, MOD13A2 V6 was used to calculate NDVI (https://lpdaac.usgs.gov/products/mod13a2v006/, accessed on 20 July 2021).

The meteorological indicators, including the land surface temperature and precipitation, were inverted by remote sensing. To describe the temperature conditions, MOD11A2 V6 was applied to determine the average 8-day land surface temperatures (LST) (https://lpdaac.usgs.gov/products/mod11a2v006/, accessed on 26 July 2020). To describe the precipitation conditions, this study used the Climate Hazards Group InfraRed Precipitation with Station data (CHIRPS), which is a quasi-global precipitation dataset (https://chc.ucsb.edu/data/chirps, accessed on 20 July 2021). 

The indicators of the land surface temperature, precipitation, and NDVI were considered during different reproductive periods of *O. d. asiaticus.* SLST, OLST, ILST, and GLST represent the land surface temperature during the spawning, overwintering, incubation, and growing periods, respectively. SP, OP, IP, and GP represent the precipitation during the spawning, overwintering, incubation, and growing periods, respectively. SNDVI, ONDVI, INDVI, and GNDVI represent the vegetation growth during the spawning, overwintering, incubation, and growing periods, respectively. VT and ST represent the vegetation type and soil type with the type numbers of 22 and 5, respectively, in the study’s region. 

We downloaded these data for each reproductive period from the Google Earth Engine (https://earthengine.google.com/, accessed on 20 July 2021). Data during the period of July 2019–July 2020 were extracted, covering the growth and reproduction periods of the *O. d. asiaticus*. The whole data were further processed following these steps: (1) the resolution of all environmental variables were either at 1 km or were resampled to 1 km by using the nearest-neighbor method; (2) the World Geodetic System 1984 benchmark was used to project all the geographic data into a geographic coordinate system. A batch processing code based on Python in ArcGIS was used to pre-process to a unified data coordinate system, spatial resolution, and data analysis range, so as to facilitate the subsequent modeling analysis.

### 2.3. MaxEnt Modeling

MaxEnt 3.4.1 [33] (biodiversityinformatics.amnh.org/open_source/maxent) was used to explore the potential distribution of *O. d. asiaticus*. As a presence-only model, MaxEnt performs well in the predictive modeling of species niches [34,35]. The general MaxEnt model formula is as follows:

(1) $$P_{w}(y|x)=\frac{1}{Z_{w}(x)}\exp\left(\sum_{i=1}^{n}w_{i}f_{i}(x,y)\right)$$

(2) $$Z_{w}(x)=\sum_{y}\exp\left(\sum_{i=1}^{n}w_{i}f_{i}(x,y)\right)$$

where *x* are the input environmental variables; *y* are the cropland locations of *O. d. asiaticus* occurrence; $f_{i}(x,y)$ is the characteristic function; $w_{i}$ is the weight of the characteristic function; *n* represents the number of datasets; and $P_{W}(y|x)$ is the output, which represents the suitability of *O. d. asiaticus* [36].

Moreover, we used the subsampling replicated run type, and 70% of the sample points were randomly selected for the training dataset, while the remaining 30% were set aside as the test dataset. The convergence threshold was set to 10^−5^. If the log loss per iteration decreased below the convergence threshold, the training would stop. The output format chosen was logistic, which represented the probability of existence (ranging between 0 and 1) [37].

### 2.4. MaxEnt Validation

We used the area under the receiver operating characteristic curve (ROC) of the test data, i.e., the AUC, to evaluate the quality of MaxEnt models. The AUC value ranges within 0–1.0. The overall accuracy of the developed model was divided into excellent (AUC > 0.9), good (0.8 < AUC ≤ 0.9), fair (0.7 < AUC ≤ 0.8), poor (0.6 < AUC ≤ 0.7), and fail (AUC ≤ 0.6) [38]. Meanwhile, the true skill statistic (TSS) was applied to validate the accuracy of the models as well. The formula of TSS is ‘sensitivity specificity −1’ and its values varied from −1 to 1. The accuracy of the model is high if the TSS value is close to 1 [39,40].

### 2.5. Data Analysis 

The environmental variables of the same category may be correlated with each other, reducing the predicted accuracy of the model [38]. The “Corrplot” package in R was used to analyze the correlation of each environmental variable. Note that the threshold value of Spearman’s rank correlation coefficients was defined as 0.8 and the factors of a high correlation were divided into different combinations.

Additionally, the percent contribution and permutation importance are the contribution and dependence (if one factor was applied alone) of each environmental variable. The high value of the percent contribution and permutation importance represent the large contribution and dependence [37].

### 2.6. Classification of Suitable Areas 

Combining the sample dataset with the optimal combination of environmental variables affecting the distributions of grasshoppers, the MaxEnt model was used to reconstruct their relationship. The existence probability (P) of grasshoppers was graded based on the expert empirical method at each grid point, where *p* < 0.05, 0.05 ≤ *p* < 0.33, 0.33 ≤ *p* < 0.66, and *p* ≥ 0.66 represent a low, moderately low, moderately high, and high suitability area, respectively [32,33].

## 3. Results

### 3.1. Accuracy Test of MaxEnt Model

**Figure 3:**
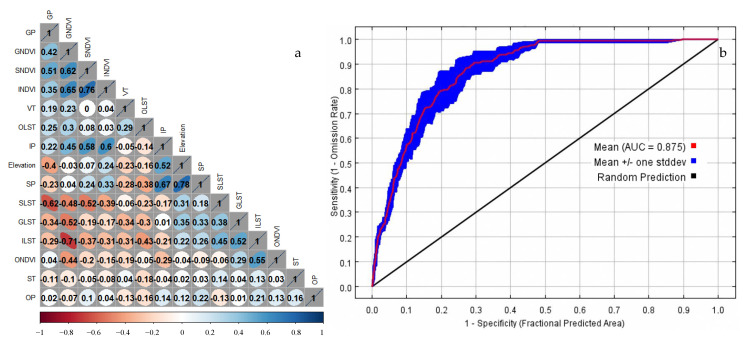
The correlation of each habitat factor (**a**) and ROC curves in MaxEnt model (**b**). The red and blue lines are the fit of MaxEnt model for training and testing data, respectively.

The correlation of all the habitat factors was calculated using the Spearman’s rank correlation coefficients to eliminate the influence of covariance on the modeling process and the interpretation of the results. The results indicated that the correlation coefficients of all the habitat factors were below 0.8, which implied that the combination of all the habitat factors can be used to develop the MaxEnt model (Figure 3a). The ROC curve of the MaxEnt model is shown in Figure 3b. The assessment indicated that the AUC value of the MaxEnt models was good, i.e., 0.875. The value of the TSS was 0.812. If the TSS value was above 0.7, the performance of the model was fair. Moreover, the TSS performed more variation among different habitat factors than the AUC.

### 3.2. Potential Suitable Areas of O. d. asiaticus

**Figure 4:**
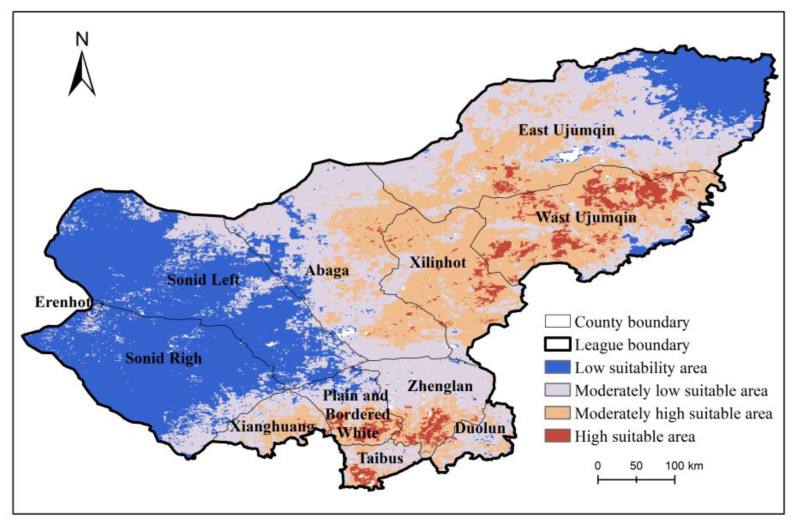
Modelling suitability of *O. d. asiaticus* occurrence by MaxEnt models. The suitability is classified into four levels: high suitability (>0.66, reddish-brown color), moderately high suitability (0.33–0.66, dark yellow color), moderately low suitability (0.05–0.33, grey color), and low suitability (<0.05, blue color).

The results indicate that the low suitability area, moderately low suitability area, moderately high suitability area, and high suitability area of Xilingol League covered the areas of 64,505 km^2^, 73,575 km^2^, 53,005 km^2^, and 7442 km^2^, respectively, accounting for 32.49%, 37.06%, 26.70%, and 3.75% of the grassland areas (Figure 4). It was worth noting that the high suitability areas were mainly distributed in Xilingol League West Urumqi, Xilinhot City, with areas of 3878 km^2^, accounting for 52.11% of the high suitability area.

### 3.3. Dominant Habitat Factors Affecting O. d. asiaticus Distribution

**Figure 5:**
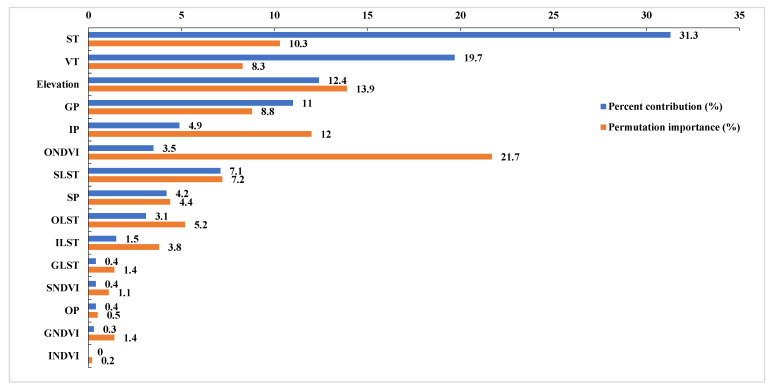
Relative percent contribution and permutation importance of habitat factors.

Figure 5 shows that the soil type (ST) had the highest percentage contribution with the value of 31.3%. The percent contributions of the vegetation type (VT), elevation, and precipitation during the growing period (GP) were above 10%. The percent contribution of the land surface temperature during the spawning period (SLST) was 7.1%. The factors with a high permutation importance were ONDVI (21.7%), elevation (13.9%), SP (12.0%), ST (10.3%), GP (8.8%), and VT (8.3%). Therefore, the six habitat factors, including ST, VT, elevation, GP, SP, and ONDVI, were selected as the main influencing factors, taking into account both the percent contribution and permutation importance.

### 3.4. Threshold Values of Dominant Habitat Factors

**Figure 6:**
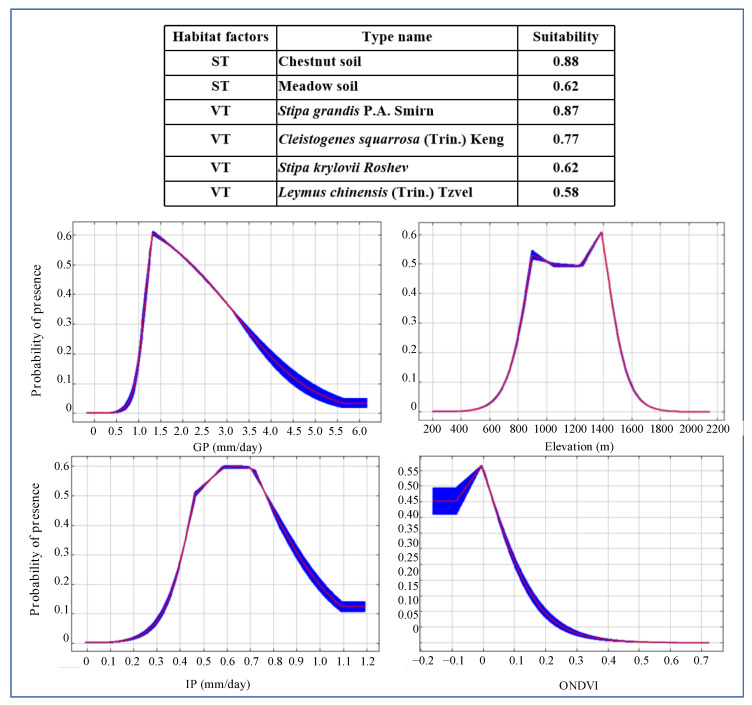
Response curves of dominant habitat factors in the model. Response curves show the relationships between existence probability of pest and the six habitat factors. For each panel, the X and Y axes represent habitat factors and probability presence. Red line represents the average of 10 replicates, and the blue margins represent standard deviation of the 10 replicates.

The response curves of dominant habitat factors are shown in Figure 6. The suitability of chestnut soil and meadow soil were higher with values of 0.88 and 0.62, respectively. The *Stipa grandis* P.A. Smirn, *Cleistogenes squarrosa* (Trin.) Keng, *Stipa krylovii Roshev,* and *Leymus chinensis* (Trin.) Tzvel were all highly suitable with values of 0.87, 0.77, 0.62, and 0.58, respectively. The elevation ranged from 800 m to 1400 m with a suitability value of above 0.5. The relationship between the suitability of *O. d. asiaticus* and precipitation was positive when the precipitation was below 1.3 mm/day during the growing period, and negative if the precipitation was over 2.0 mm/day. However, the precipitation during 1.3–2.0 mm/day was the most suitable. In addition, the suitability of *O. d. asiaticus* increased when the mean precipitation during the incubation period was less than 0.5 mm/day, while the suitability decreased when the value of the precipitation was over 0.7 mm/day. The suitability is high when the ONDVI was between −0.1 and 0.1. The relationship between the suitability of *O. d. asiaticus* and ONDVI during the overwintering period was positive when the value of ONDVI was below 0, while the suitability decreased when the value of ONDVI was over 0.

## 4. Discussion

Remote sensing data enable the possibility of real-time monitoring, allowing short-term predictions of *O. d. asiaticus* distribution. This study used MODIS remote sensing products from assimilation systems as the main data sources of the environmental factors from September 2019 to June 2020 in Xilingol League. The addition, the remote sensing data improved the spatial and temporal resolutions of the potential distributions and increased the accuracy of the simulated results. To date, remote sensing has been widely applied in monitoring plant diseases and pests [41]. Furthermore, remote sensing will be even more effective in the prevention and control of *O. d. asiaticus* in the coming decades. For example, it will be used to monitor the severity of grassland disasters caused by pests based on the changes in forage biomass. Meanwhile, it will be applied to narrow the scope of the control through a remote sensing inversion of the host plants, further improving the precision of the preventative measures. Currently, the grassland grazing activities by pastoralists resulted in a large variability in the growing periods of the pastures. Likewise, cloud and foggy weather frequently interfered with the coefficient of crop remote sensing inversion in Xilingol League. Under these circumstances, the vegetation index, as a relatively convenient and efficient method, can reflect the vegetation conditions and improve the accuracy.

This study showed that the majority of potentially habitable areas for grasshoppers were distributed in West Urumqi, Xilinhot City, East Urumqi, Abaga Banner, and Xianghuang Banner of Xilingol League. This analysis also identified the key habitat factors determining the suitability for grasshoppers. To comprehensively consider the impact of the environmental factors, this work used a total of six environmental factors from three categories, including meteorologic, vegetation, and soil. However, most previous studies only considered the impact of meteorological factors on the suitability for *O. d. asiaticus* [26,42,43]. Although meteorology is a vital factor affecting the suitability for *O. d. asiaticus*, the influences of vegetation and soil cannot be ignored.

The temperature, texture, moisture content, and salt content of soil influence the growth and reproduction of grasshoppers in grasslands [2]. The physical and chemical properties vary greatly among the different soil types. Here, we found that the percentages of affecting the *O. d. asiaticus* distribution for the soil and vegetation were 31.3% and 19.7%, respectively. Both the type and composition of vegetation affected the *O. d. asiaticus* feeding. In addition, the growth of vegetation affects the near-surface hydrothermal conditions, which ultimately affects the survival and development of *O. d. asiaticus*. A previous study indicated that needlegrass and sheepgrass survived well in warm and dry climatic conditions, which agreed with the results of our study [2]. Additionally, differences in temperature and precipitation, especially the temperature associated with different elevations, have a significant impact on the occurrence of *O. d. asiaticus* because it affects its growth and development [19]. In the study region, the difference in the elevation is minor; therefore, it is not the key factor affecting the *O. d. asiaticus* distribution.

Moreover, the land surface temperature, precipitation, and NDVI factors were divided into four periods according to the different growth stages of grasshoppers in this region. We concluded that the highest suitability for *O. d. asiaticus* was when the ONDVI value was less than 0. This is because an ONDVI value of less than 0 represents that there is a snow cover [44]. The mean precipitation during the growing period (GP) is of great importance for *O. d. asiaticus*, directly affecting the *O. d. asiaticus* growth, changing the grassland vegetation to indirectly influence its growth. We manifest that GP accounted for 11.0% among the whole environmental factors. The relationship between the suitability of *O. d. asiaticus* and the precipitation was positive when the precipitation was below 1.3 mm/day during the growing period, and negative if the precipitation was over 2.0 mm/day. Meanwhile, GP with the value of 1.3–2.0 mm/day was most suitable for *O. d. asiaticus*. The reason is that the hatching rate of *O. d. asiaticus* was reduced when they were immersed in warm and wet soil for a long period, which was consistent with other studies [44,45]. Therefore, the impact of precipitation on the *O. d. asiaticus* distribution is higher than the other climatic factors.

In general, this study only extracted a suitable distribution using grasshopper occurrence data from a short time series; thus, this research could provide methodological support for an early warning and the efficient prevention and control of *O. d. asiaticus*. Certainly, a further data collection should follow to improve the accuracy of the future modeling predictions. Furthermore, the selection of influence factors was limited, and other factors, such as the grasshopper control situation, grazing situation, and the migration of grasshoppers from external areas, are not considered. It is well known that the modeling precision with species distribution model ensembles is better than a single species distribution model due to the uncertainties of each model. Therefore, the next step is to predict suitable grasshopper areas with multispecies distribution models.

## Figures and Tables

**Table 1:** Environmental variables for modeling the suitability of *O. d. asiaticus*.

Category	Variables	Abbreviation	Resolution	Units	Data Source
Soil	Soil type	ST	1:1 million		China soil map
Topography	Elevation	Elevation	30 m	m	ASTER GDEM V3
Vegetation	Vegetation type	VT	1:1 million		China vegetation map
	Normalized difference vegetation index	NDVI	1000 m		MOD13A2 V6 product
Meteorology	Land surface temperature	LST	1000 m	°C	MOD11A2 V6 product
	Precipitation	P	0.05 degrees	mm/day	Climate Hazards Group InfraRed Precipitation

## Data Availability

The data presented in this study are available on request from the corresponding author. The data are not publicly available because the data need to be used in future work.

## References

[1] Marion L.G., Rick O., Arianne C. (2019). A global review on locusts (*Orthoptera: Acrididae*) and their interactions with livestock grazing practices. Front. Ecol. Evol..

[2] Du G., Zhao H., Tu X. (2018). Division of the inhabitable areas for *Oedaleus decorus asiaticus* (Bey-Bienko) in Inner Mongolia. Plant Prot..

[3] Li G., Kong D., Li F., Liu Q., Wang H. (2017). Reviews and prospects on studies of locust breeding area evolution and drainage network change in China during the historical period. Trop. Geogr..

[4] Shi W., Tan S. (2019). Current status and trend on grasshopper and locust biological control. Chin. J. Biol. Control..

[5] Chen J., Wang X. (2012). Progress in application of remote sensing and GIS to the study of locust habitats. Ecol. Environ. Sci..

[6] Wang H., Yu F., Hu H., Ji R. (2014). Climatic changes in suitable distribution areas of *Calliptamus italicus* L.. Chin. J. Agrometeorol..

[7] Waldner F., Ebbe M.A.B., Cressman K., Defourny P. (2015). Operational monitoring of the desert locust habitat with earth observation: An assessment. ISPRS Int. J. Geo. Inf..

[8] Xing W., Pang B., Hao S. (2017). The combined effects of livestock grazing and seasonally increasing precipitation on the development and survival of *Dasyhippus barbipes* (Fischer-Waldheim) in Inner Mongolia. Chin. J. Appl. Entomol..

[9] Wang B., Edward D., Cathy W., Waters C., Spessa A., Lawton D., Feng P.Y., Liu D.L. (2019). Future climate change likely to reduce the Australian Plague Locust (*Chortoicetes Terminifera*) seasonal outbreaks. Sci. Total Environ..

[10] Propastin P. (2013). Satellite-based monitoring system for assessment of vegetation vulnerability to locust hazard in the River Ili delta (Lake Balkhash, Kazakhstan). J. Appl. Remote Sens..

[11] Stige L.C., Chan K.S., Zhang Z., Frank D., Stenseth N.C. (2007). Thousand-year-long Chinese time series reveals climatic forcing of decadal locust dynamics. Proc. Natl. Acad. Sci. USA.

[12] Tian H., Stige L.C., Cazelles B., Kausrud K.L., Svarverud R., Stenseth N.C., Zhang Z. (2011). Reconstruction of a 1,910-y-long locust series reveals consistent associations with climate fluctuations in China. Proc. Natl. Acad. Sci. USA.

[13] Chen J., Sheng S., Wang W. (2014). Study on effect of type of locust habitats on locust plague based on multi-temporal Landsat TM Data. J. Ecol. Rural. Environ..

[14] Clissold F.J., Simpson S.J. (2015). Temperature, food quality and life history traits of herbivorous insects. Curr. Opin. Insect Sci..

[15] Deveson E. (2013). Satellite normalized difference vegetation index data used in managing Australian plague locusts. J. Appl. Remote Sens..

[16] Renier C., Waldner F., Jacques D.C., Ebbe M.A.B., Cressman K., Defourny P. (2015). A dynamic vegetation senescence indicator for near-real-time desert locust habitat monitoring with MODIS. Remote Sens..

[17] Latchininsky A.V. (2013). Locusts and remote sensing: A review. J. Appl. Remote Sens..

[18] Vallebona C., Crisci A., Vecchi A.D., Genesio G., Maracchi, Pasqui M. West Africa Desert Locust Infestations: Connections with Regional Atmospheric Circulation Patterns. Proceedings of the 20th Conference on Climate Variability and Change.

[19] Ni S. (2002). Remote Sensing Monitoring and Prediction of Grasshoppers in the Area around Qinghai Lake.

[20] Nishide Y., Suzuki T., Tanaka S. (2017). The hatching time of *Locusta migratoria* under outdoor conditions: Role of temperature and adaptive significance. Physiol. Entomol..

[21] Bernays E.A., Gonzalez N., Angel J., Bright K.L. (1995). Food mixing by generalist grasshoppers: Plant secondary compounds structure the pattern of feeding. J. Insect Behav..

[22] Branson D.H. (2008). Influence of a large late summer precipitation event on food limitation and grasshopper population dynamics in a northern great plains grassland. Environ. Entomol..

[23] Estes L.D., Bradley B.A., Beukes H., Hole D.G., Lau M., Oppenheimer M.G., Schulze R., Tadross M.A., Turner W.R. (2013). Comparing mechanistic and empirical model projections of crop suitability and productivity: Implications for ecological forecasting. Global Ecol. Biogeogr..

[24] Lozano F.J., Suarez-Seoane S., Kelly M., Luis E. (2008). Multi-scale approach for modeling fire occurrence probability using satellite data and classification trees: A case study in a mountainous Mediterranean region. Remote Sens. Environ..

[25] Farashi A., Kaboli M., Karami M. (2013). Predicting range expansion of invasive raccoons in northern Iran using ENFA model at two different scales. Ecol. Inform..

[26] Le Z., Bai Y., Liu L. (2013). The effect of temperature on hatching of Asiatic migratory locust in the grassland of north-east China. J. Meteorol. Environ..

[27] Adriaansen C., Woodman J.D., Deveson E., Drake V. (2016). The Australian Plague Locust-Risk and Response. Biological and Environmental Hazards, Risks, and Disasters.

[28] Crooks W.T.S., Cheke R.A. (2014). Soil moisture assessments for brown locust *Locustana pardalina* breeding potential using synthetic aperture radar. J. Appl. Remote Sens..

[29] Zhang X., Rao J., Pan Y. (2015). Progressive approach for risk prediction of rangeland locust hazard in Xinjiang based on remotely sensed data. Trans. Chin. Soc. Agric. Eng..

[30] Löw F., Waldner F., Latchininsky A., Biradar C., Bolkart M., Colditz R.R. (2016). Timely monitoring of Asian Migratory locust habitats in the Amudarya delta, Uzbekistan using time series of satellite remote sensing vegetation index. J. Environ. Manage..

[31] Cissé S., Ghaout S., Babah M.A., Kamara S., Piou C. (2016). Field verification of the prediction model on desert locust adult phase status from density and vegetation. J. Insect Sci..

[32] Lu L., Sun Z., Eerdeng Q., Ye H., Huang W., Nie C., Wang K., Zhou Y. (2022). Using remote sensing data and species- environmental matching model to predict the potential distribution of grassland rodents in the northern China. Remote Sens..

[33] Manning M. (2006). The treatment of uncertainties in the fourth IPCC assignment report. Adv. Clim. Chang. Res..

[34] Kumar S., Neven L.G., Zhu H., Zhang R. (2015). Assessing the global risk of establishment of *Cydia pomonella* (Lepidoptera: Tortricidae) using CLIMEX and MaxEnt niche models. J. Econ. Entomol..

[35] Zingore K.M., Sithole G., Abdel-Rahman E.M., Mohamed S.A., Ekesi S., Tanga C.M., Mahmoud M.E.E. (2020). Global risk of invasion by *Bactrocera zonata*: Implications on horticultural crop production under changing climatic conditions. PLoS ONE.

[36] Ning S., Wei J., Feng J. (2017). Predicting the current potential and future world wide distribution of the onion maggot, *Delia antiqua* using maximum entropy ecological niche modeling. PLoS ONE.

[37] Huang Y., Dong Y., Huang W., Ren B., Deng Q., Shi Y., Bai J., Ren Y., Geng Y., Ma H. (2020). Overwintering distribution of fall armyworm (*Spodoptera frugiperda*) in Yunnan, China, and influencing environmental factors. Insects.

[38] Phillips S.J., Anderson R.P., Schapire R.E. (2006). Maximum entropy modeling of species geographic distributions. Ecol. Model.

[39] Allouche O., Tsoar A., Kadmon R. (2006). Assessing the accuracy of species distribution models: Prevalence, kappa and the true skillstatistic (TSS). J. Appl. Ecol..

[40] Frederico R.G., De Marco P.J., Zuanon J. (2014). Evaluating the use of macroscale variables as proxies for local aquatic variables and to model stream fish distributions. Freshw. Biol..

[41] Zhang J., Huang Y., Pu R., Gonzalez-moreno P., Yuan L., Wu K., Huang W. (2019). Monitoring plant diseases and pests through remote sensing technology: A review. Comput. Electron. Agric..

[42] Guo A., Wang J., Wang C. (2009). Meteorological suitability index of grasshopper growth and development in Inner Mongolia. Meteorol. Sci. Technol..

[43] Bai Y., Liu L., Wu L. (2007). The relationship between the occurrence of locusts and the characteristics of atmospheric circulation in Inner Mongolia. Chin. J. Ecol..

[44] Bai Y., Liu L., Gao S. (2013). Study on Meteorological Monitoring and Forecasting of Grassland Locust and Countermeasures.

[45] Sillero N. (2011). What does ecological modelling model? A proposed classification of ecological niche models based on their underlying methods. Ecol. Model.

